# Parental Genome Imbalance Causes Post-Zygotic Seed Lethality and Deregulates Imprinting in Rice

**DOI:** 10.1186/s12284-016-0115-4

**Published:** 2016-08-27

**Authors:** Hong-yu Zhang, Ming Luo, Susan D. Johnson, Xiao-wei Zhu, Lei Liu, Fang Huang, Yu-tong Liu, Pei-zhou Xu, Xian-jun Wu

**Affiliations:** 1 Rice Research Institute, Sichuan Agricultural University, Wenjiang Chengdu, People’s Republic of China; 2 Commonwealth Scientific and Industrial Research Organization (CSIRO), Agriculture Flagship, PO Box 1600, Canberra, 2601 ACT Australia; 3 Commonwealth Scientific and Industrial Research Organization (CSIRO), Agriculture Flagship, Waite Campus, PMB 2, Glen Osmond, SA 5064 Australia; 4 Institute of genetics and developmental biology, Chinese Academy of Sciences, 100101 Beijing, China

**Keywords:** Polyploid, Endosperm, Embryo, Cellularization, Reproduction, Polycomb, DNA methylation, Imprinting, Triploid block

## Abstract

**Background:**

Reproductive isolation between rice of different ploidy levels is manifested as endosperm and embryo abortion in seeds produced by interploidy crosses. Genomic imprinting is considered to be the underlying mechanism establishing the post-zygotic hybridization barrier. We characterized disrupted seed development in reciprocal crosses between a diploid Japonica rice and a tetraploid Indica rice.

**Results:**

Triploid seeds from these crosses had aborted development and could not germinate in soil but could be rescued in culture medium with significantly more seeds developing to seedlings in the 4n × 2n (♀-♂) cross with excess maternal genomes than in the 2n × 4n cross with excess paternal genome. Consistent with previous findings, precocious endosperm cellularization and bigger embryos were observed in the seeds from the maternal excess cross, whereas absence of cellularization and arrested globular embryos were found in the seeds from the paternal excess cross, supporting the idea that endosperm cellularization is an important transition for embryo development. Moreover, we found that starch granules were persistently deposited in the pericarp parenchyma cells of the paternal excess cross, while pericarp starch gradually decreased and relocated to the developing endosperm in balanced and maternal excess crosses in which cellularization and starch deposition occur in endosperm, suggesting that parental genome balance influences pericarp starch relocation via cellularization and starch deposition. Loss of imprinting, or altered expression of imprinted genes and epigenetic regulators, OsFIE2 and OsMET1b were observed, implying the potential role of imprinting and epigenetic mechanisms in regulating the differential parental genome dosage effects on endosperm development.

**Conclusions:**

Our results support the hypothesis that the maternal genome dosage promotes endosperm cellularization and the paternal genome dosage delays or inhibits cellularization via contributing different sets of imprinted genes.

**Electronic supplementary material:**

The online version of this article (doi:10.1186/s12284-016-0115-4) contains supplementary material, which is available to authorized users.

## Background

Crosses between two parents with different ploidy levels result in a change of parental genome ratios by adding either additional maternal or paternal copies to the embryo and endosperm. Additionally, absolute ploidy levels and the relative genome dosages of the two fertilization products to the maternal tissues are also altered (Table 1). The consequences of interploidy hybridization on seed development have been studied in a wide range of species (Watkins 1932; Howard 1939; Brink and Cooper 1947; Hakansson 1956; Nishiyama and Inomata 1966; Lin 1984; Ramsey and Schemske 1998; Scott et al. 1998; Gutierrez-Marcos et al. 2003; Stoute et al. 2012; Kradolfer et al. 2013a; Sekine et al. 2013). Endosperm development is more affected than embryo and shows contrasting phenotypes between reciprocal interploidy crosses: with precocious cellularization and poor proliferation occurring in the maternal excess cross, and delayed or interrupted cellularization and over proliferation occurring in the paternal excess cross in each species. This suggests that similar mechanisms operate in different species to regulate post-zygotic incompatibility between plants of different ploidy levels. The analysis of the crosses between maize 2n or 4n pollen donors and the 2n seed parent *indeterminate gametophyte* which gives varying numbers of polar nuclei to the endosperm suggested that a 2maternal (m):1paternal (p) genomic ratio rather than a specific ploidy level of endosperm is critical for normal seed development (Lin 1984).

**Table 1:** Ploidy levels and parental genome ratios of rice seed components, and seed setting and embryo rescue rates from balanced and unbalanced crosses

Crosses	Maternal ploidy	Embryo ploidy (m:p)	Endosperm ploidy (m:p)	Seed setting rate	Embryo rescue rate
Nip2n × Nip2n	2n	2n (1:1)	3n (2:1)	27.42 % (164/598)	30.43 % (42/138)
TH4n × TH4n	4n	4n (2:2)	6n (4:2)	14.04 % (183/1303)	37.35 % (31/83)
TH4n × Nip2n	4n	3n (2:1)	5n (4:1)	17.26 % (231/1338)	48.86 % (86/176)
Nip2n × TH4n	2n	3n (1:2)	4n (2:2)	10.05 % (255/2537)	4.69 % (16/341)

The 2 m:1p requirement was believed to be an indication of existence of genomic imprinting in endosperm (Haig and Westoby 1989; 1991). Genomic imprinting (or imprinting) refers to the selective expression of a parental copy of a gene depending on its parent-of-origin due to the differential epigenetic modifications of parental genomes. It has been hypothesized that maternally expressed genes (MEGs) and paternally expressed imprinted genes (PEGs) have differential functions in regulate endosperm development (Haig and Westoby 1991; Scott et al. 1998). In plants, imprinted genes are predominantly found in endosperm but not in embryo (Luo et al. 2011; Waters et al. 2011; Wolff et al. 2011). Over dosage of MEGs in maternal excess cross and PEGs in the paternal excess cross are thought to contribute to the contrasting endosperm phenotypes between reciprocal interploidy crosses (Haig and Westoby 1991; Scott et al. 1998). Thus imprinting provides a likely explanation for the parent-of-origin specific effects on endosperm development in interploidy crosses. Several hypotheses have been proposed to explain the evolution and function of imprinting and its role in interploidy cross (Haig and Westoby 1989; Birchler 1993; Dilkes and Comai 2004; Josefsson et al. 2006; Jullien and Berger 2010; Berger et al. 2012).

Dicot Arabidopsis has been used as model to analyse the parental genome imbalance on seed development and the underlying molecular mechanisms. In reciprocal crosses using 2n and 4n in certain ecotypes or using 2n and 6n, embryos arrest with an undisturbed basic body plan but accompanied with precocious endosperm cellularization or discrupted cellularization based on the directions of the crosses (Scott et al. 1998). The FERTILIZATION INDEPENDENT SEED (FIS) Polycomb Repressive Complex (PRC2) complex which catalyzes histone H3 lysine 27 trimethylation (H3K27me) and maintenance DNA methyltransferase 1 (MET1) play essential and antagonistic roles in regulating endosperm cellularization via controlling expression of key imprinted genes in Arabidopsis (Luo et al. 2000; Adams et al. 2000; Erilova et al. 2009; Kradolfer et al. 2013a; 2013b; Schatlowski et al. 2014). Seed defects or altered cellularization in the maternal excess cross or in the paternal excess cross are partially suppressed by the loss of function of a maternally expressed imprinted gene (MEG) *PRC2 MEDEA* (*MEA*), or by a paternally derived DNA hypomethylated genome, respectively (Erilova et al. 2009; Kradolfer et al. 2013a; Schatlowski et al. 2014), suggesting a connection between interploidy seed defects and deregulated function of PRC2 and MET1. Loss of imprinting and/or altered expression at *MEA*, *FIS2* and *PHERES 1* loci have been observed in interploidy crosses (Erilova et al. 2009; Jullien and Berger 2010). The connection between loss of imprinting and endosperm abortion is yet to be investigated.

In the monocot rice, differential parental genome dosage effects on seed development in the reciprocal crosses between a 2n Japonica line and its own 4n line have recently been observed (Sekine et al. 2013). Endosperm development is inhibited with precocious cellularization but accompanied by swollen embryos in the 4n × 2n maternal excess cross, while seeds in the reciprocal paternal excess crosses display disrupted cellularization with arrested globular embryos. Starch granules are not seen in the disrupted endosperm of the 2n × 4n cross, suggesting cellularization is a prerequisite for the starch accumulation in endosperm. It has been implied that transitory pericarp starch in parenchyma cells plays an important role in seed development, as this type of starch is broken down and relocated to developing seed (Duffus and Rosie 1973; Sato 1984; Caley et al. 1990). It remains to be determined how relocation of the pericarp starch responds to the altered cellularization and starch deposition in endosperm with parental genome imbalance.

The rice genome contains two *FERTILISATION INDEPENDENT ENDOSPERM* (*FIE*) genes encoding members of the PRC2 complex (Luo et al. 2009; Nallamilli et al. 2013; Li et al. 2014) and two MET1 genes (Yamauchi et al. 2009; Hu et al. 2014). *OsFIE2* and *OsMET1b* are the major genes potentially regulating endosperm development and other processes (Luo et al. 2009; Yamauchi et al. 2009; Nallamilli et al. 2013; Hu et al. 2014; Li et al. 2014). Given that PRC2 and MET1 are required for establishing postzygotic hybridization barrier via controlling imprinted genes in Arabidopsis (Erilova et al. 2009; Kradolfer et al. 2013a; Schatlowski et al. 2014) and differential expression of imprinted genes between parental genomes in rice are likely controlled by differential H3K27me and DNA methylation (Rodrigues et al. 2013; Du et al. 2014), it is interesting to know if the rice PRC2 and MET1 counterparts have the same functions in regulating postzygotic hybridization barrier as in Arabidopsis, and whether loss of imprinting also occurs in the rice endosperm with parental genome imbalance.

Subspecies hybridization between Indica and Japonica rice at 2n gives viable seeds with no apparent endosperm defect (Luo et al. 2011). In this study, we carried out a histological study on the seeds derived from reciprocal crosses between a 2n Japonica rice and an Indica 4n rice. We show similar patterns of disruption of endosperm and embryo development to the seeds from the interploidy reciprocal crosses in the Japonica background (Sekine et al. 2013). We observed that the deposition of starch granules in the pericarp parenchyma cells were different in the paternal excess cross compared with those in other crosses. With the sequence polymorphisms between the two parents, we were able to analyze the status of imprinted gene expression and observed that several imprinted genes lost imprinting or expression, along with the altered expression of two potential imprinting regulators, the *PRC2* gene *OsFIE2* and the *MET1* ortholog *OsMET1b* in the parental genome unbalanced endosperm. This study provides useful clues for further understanding the mechanisms underlying reproductive isolation between rice of different ploidy levels.

## Results

### Seed Development is Perturbed in the Reciprocal Interploidy Crosses

Using a diploid Japonica cultivar Nipponbare (Nip2n) and a tetraploid Indica line Tetra-Haitian (TH4n), we performed both self- and cross-pollinations to examine the effects of parental genome imbalance on seed development. We used the root tips to count the chromosomes to confirm ploidy levels for both parents and the progeny (Additional file 1: Figure S1). Panicles from the crosses, TH4n × TH4n, Nip2n × Nip2n, Nip2n × TH4n (paternal excess), and TH4n × Nip2n (maternal excess) were harvested for analysis of seed set, viability and development.

There was a difference in the rates of seed setting among the four sets of crosses after cross-pollination, with crosses involving a 4n parent showing lower seed set (Table 1). All the seeds were aborted in both unbalanced crosses at maturity (Additional file 1: Figure S1). The outcomes of TH4n reciprocally crossed to H2n, the 2n progenitor line of TH2n were similar to the interploidy crosses between subspecies (Additional file 1: Figure S1E) and the Nip2n crossed with H2n Indica gave normal seeds, suggesting that seed abortion was not due to the difference of genetic background between Japonica and Indica but specific to the unbalanced crosses.

Seeds derived from the rice unbalanced crosses could not be germinated on soil but the developing embryos were able to be rescued on nutrient-enriched medium. We rescued developing embryos at days 5, 8, 11, and 14 post-pollination on MS media, with an average of 48.86 % embryos in TH4n × Nip2n and 4.69 % embryos in Nip2n × TH4n being rescued (Table 1). The resulting seedlings reached maturity without any obvious developmental abnormality, except for the sterility expected from a 3n rice plant. Root tip chromosome counting confirmed the seedlings from the unbalanced crosses were triploid (Additional file 1: Figure S1C and D). Interestingly, embryos from the maternal excess TH4n × Nip2n cross had a much higher germination rate than those from the paternal excess Nip2n × TH4n cross, suggesting that postzygotic hybridization barriers between the two unbalanced reciprocal crosses are asymmetric and embryos are less affected by adding extra maternal genome copies.

### Distinct Endosperm and Embryo Development Patterns Result from the Maternal and Paternal Excess Crosses

**Fig. 1:**
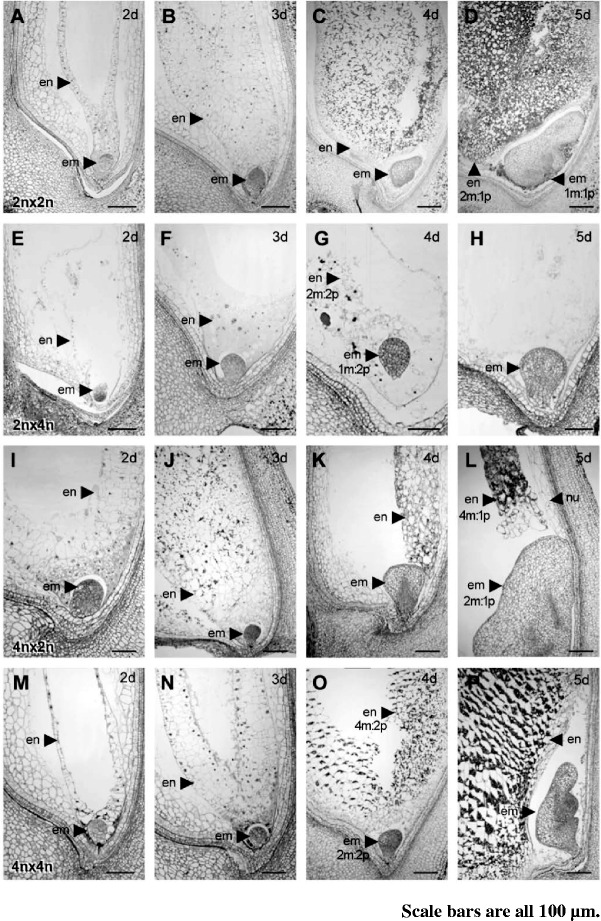
Sections of developing rice seeds in parental genome balanced and unbalanced crosses showing endosperm and embryo development. **a**-**d** Sections of Nip2n × Nip2n developing seeds at 2 DAP **a**, 3 DAP **b**, 4 DAP **c**, and 5 DAP **d**. Endosperm (en) and embryo (em) are indicated by arrows. Endosperm is not cellularized until 3 DAP. Starch granules (black dots) can be seen in the cellularized endosperm. **e**-**h** Sections of Nip2n × TH4n developing seeds at 2 DAP **e**, 3 DAP **f**, 4 DAP **g**, and 5 DAP **h**. Endosperm is never cellularized. Embryo arrests at globular stage. **i**-**l** Sections of TH4n × Nip2n developing seeds at 2 DAP **i**, 3 DAP **j**, 4 DAP **k**, and 5 DAP **l**. Precocious cellularization of endosperm occurs at 2 DAP and stop growing at 4 DAP. Embryo appears to be enlarged at 5 DAP. **m**-**p** Sections of TH4n × TH4n developing seeds at 2 DAP **m**, 3 DAP **n**, 4 DAP **o**, and 5 DAP **p**. Development of endosperm and embryo is similar to that in Nip2n × Nip2n

To understand the effects of parental genome imbalance on seed development, developing seeds from the balanced and unbalanced crosses were sectioned at 2, 3, 4 and 5 DAP (days after pollination). The development of embryo and endosperm in the Nip2n × Nip2n cross followed the same pattern as reported previously (Fig. 1a–d; Brown et al. 1996; Itoh et al. 2005). At 2 DAP, the embryo was at the globular stage (Fig. 1a). The endosperm was a syncytium, containing nuclei lining the entire peripheral cytoplasm, with a large central cavity. At 3 DAP, the embryo was slightly elongated and endosperm cellularization was well under way. The division of the endosperm cells was rapidly progressing toward the centre of the embryo sac, leaving a smaller cavity (Fig. 1b). By 4 DAP, the embryo differentiated a coleoptile primordium and accumulation of starch granules could be seen in the endosperm cells (Fig. 1c). At 5 DAP, the embryo differentiated further and a significant number of starch granules had accumulated in the endosperm (Fig. 1d). Seed development observed in another balanced cross (Fig. 1m–p), TH4n × TH4n, was similar to that in the Nip2n × Nip2n cross. By 2 DAP, the embryo was globular and the endosperm was uncellularized (Fig. 1m). Similarly, at 3 DAP, the peripheral endosperm had cellularized (Fig. 1n). The development of embryo and endosperm at later stages (TH4n × TH4n) followed the same patterns as seen in the Nip2n × Nip2n seeds (Fig. 1o and p).

The seed derived from the paternal excess cross (Nip2n × TH4n) showed a different pattern of development. At 2 DAP, the seed contained a globular embryo and syncytial endosperm similar to the seed from the balanced crosses (Fig. 1e). However, the embryo arrested at the globular stage (Fig. 1e–h). Endosperm nuclei may have undergone additional divisions but remained uncellularized. At 5 DAP, endosperm nuclei were no longer visible and the embryo sac was full of clear liquid. No starch granules were observed in the endosperm at any stages in the paternal excess cross.

**Fig. 2:**
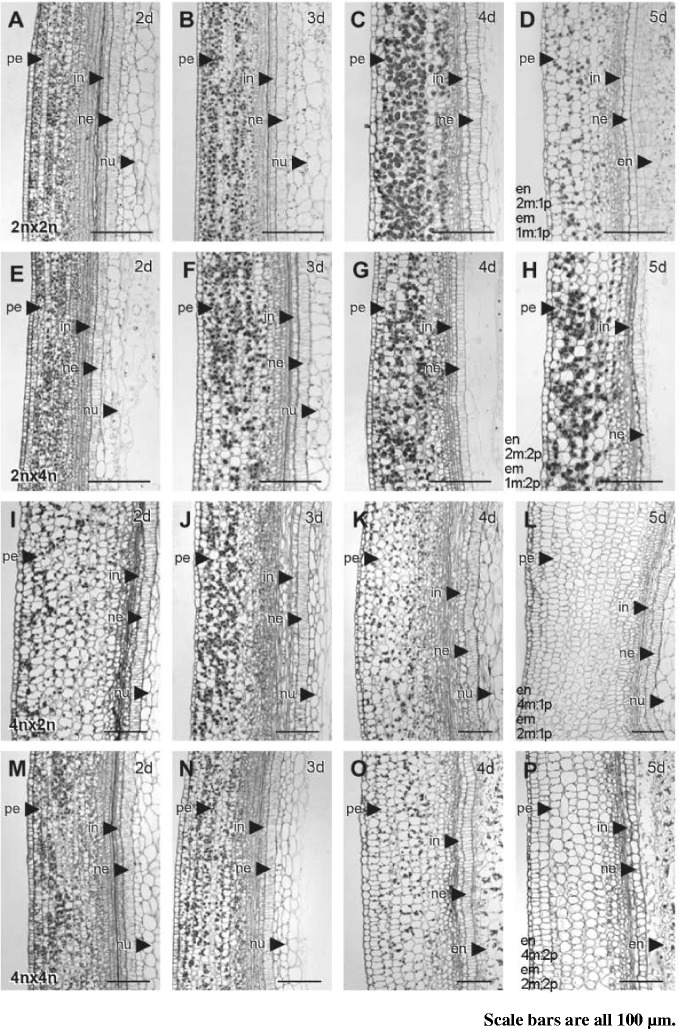
Sections of developing rice seeds in parental genome balanced and unbalanced crosses showing deposition of starch grains in pericarp cells. **a**-**d** Sections of Nip2n × Nip2n developing seeds focusing on pericarp at 2 DAP **a**, 3 DAP **b**, 4 DAP **c**, 5 DAP **d**. pericarp (pe), integument (in), nucellar epidermis (ne) and nucellus (nu) are indicated by arrows. **e**-**h** Sections of Nip2n × TH4n developing seeds focusing on pericarp at 2 DAP **e**, 3 DAP **f**, 4 DAP **g**, 5 DAP **h**. **i**-**l** Serial sections of TH4n × Nip2n developing seeds focusing on pericarp at 2 DAP **i**, 3 DAP **j**, 4 DAP **k**, and 5 DAP **l**. **m**-**p** Serial sections of TH4n × TH4n developing seeds focusing on pericarp at 2 DAP **m**, 3 DAP **n**, 4 DAP **o**, and 5 DAP **p**

The seed derived from the maternal excess cross (TH4n × Nip2n) followed a developmental pattern different from that seen in the above crosses (Fig. 1i–l). By 2 DAP, the endosperm had already cellularized, whereas the embryo was at the globular stage, thus the onset of cellularization in the maternal excess cross was earlier than that in the balanced cross (Fig. 1i). At 3 DAP, starch grains became obvious in cellularized endosperm (Fig. 1j). By 4 and 5 DAP, the endosperm apparently stopped growing but pericarp continued to expand, leaving the arrested endosperm detached from pericarp. The embryos at 5 DAP appeared to be enlarged with a swollen scutellum (Fig. 1k and l). Compared with TH4n × TH4n, the TH4n × Nip2n showed delayed degeneration of the nucellus (Fig. 2l).

In summary, seed development between the two balanced crosses is similar, following the patterns as previously described (Brown et al. 1996; Itoh et al. 2005). In the maternal excess cross, endosperm was precociously cellularized and stopped growing in early seed development stage, with embryos appearing enlarged and persistent nucellus. However, in the paternal excess cross, the embryo arrested at the globular stage and the endosperm remained uncellularized and became aborted. The developmental patterns of embryo and endosperm in the interploidy crosses in this study are similar to those observed in a study of interploidy crosses in the Japonica background (Sekine et al. 2013).

### Pericarp Starch Granules in Interploidy Crosses

Starch granules in the pericarp of a developing seed are relocated to rapidly growing endosperm tissues thus serving as temporary starch storage in cereals (Caley et al. 1990; Duffus and Rosie 1973; Sato 1984). The contrasting outcomes of endosperm and embryo development, as well as starch accumulation between interploidy crosses, prompted us to investigate the developmental patterns of starch granule deposition in the pericarp. The pericarp starch granules located in parenchyma cells accumulated in a similar manner up to 4 DAP in both the Nip2n × Nip2n and Nip2n × TH4n seeds (Fig. 2a–c and e–g). However, at 5 DAP when the Nip2n × Nip2n seed was rapidly expanding and accumulating starch in the cellularized endosperm (Fig. 1d), the number of starch granules decreased in the pericarp (Fig. 2d). In contrast, the number of starch granules remained unchanged in the pericarp of the Nip2n × TH4n seed (Fig. 2h) where no starch accumulation in the abortive endosperm was observed (Fig. 1h). There was no obvious difference in accumulation of pericarp starch during seed development between the TH4n × Nip2n and TH4n × TH4n crosses (Fig. 2i–p), where both underwent endosperm cellularization (Fig. 1i–p). The starch granules almost completely disappeared in the pericarp parenchyma cells in the TH4n pollinated with either Nip2n or TH4n at 5 DAF (Fig. 2l and p). This suggests that the translocation of the transitory pericarp starch into endosperm is affected by the status of endosperm development with cellularization and/or starch accumulation being the signal for translocation of starch from pericarp, possibly via a feedback mechanism.

### Expression of PRC2 Gene *OsFIE2* and DNA Methyltransferase Gene *OsMET1b* in the Endosperm of Interploidy Crosses

**Fig. 3:**
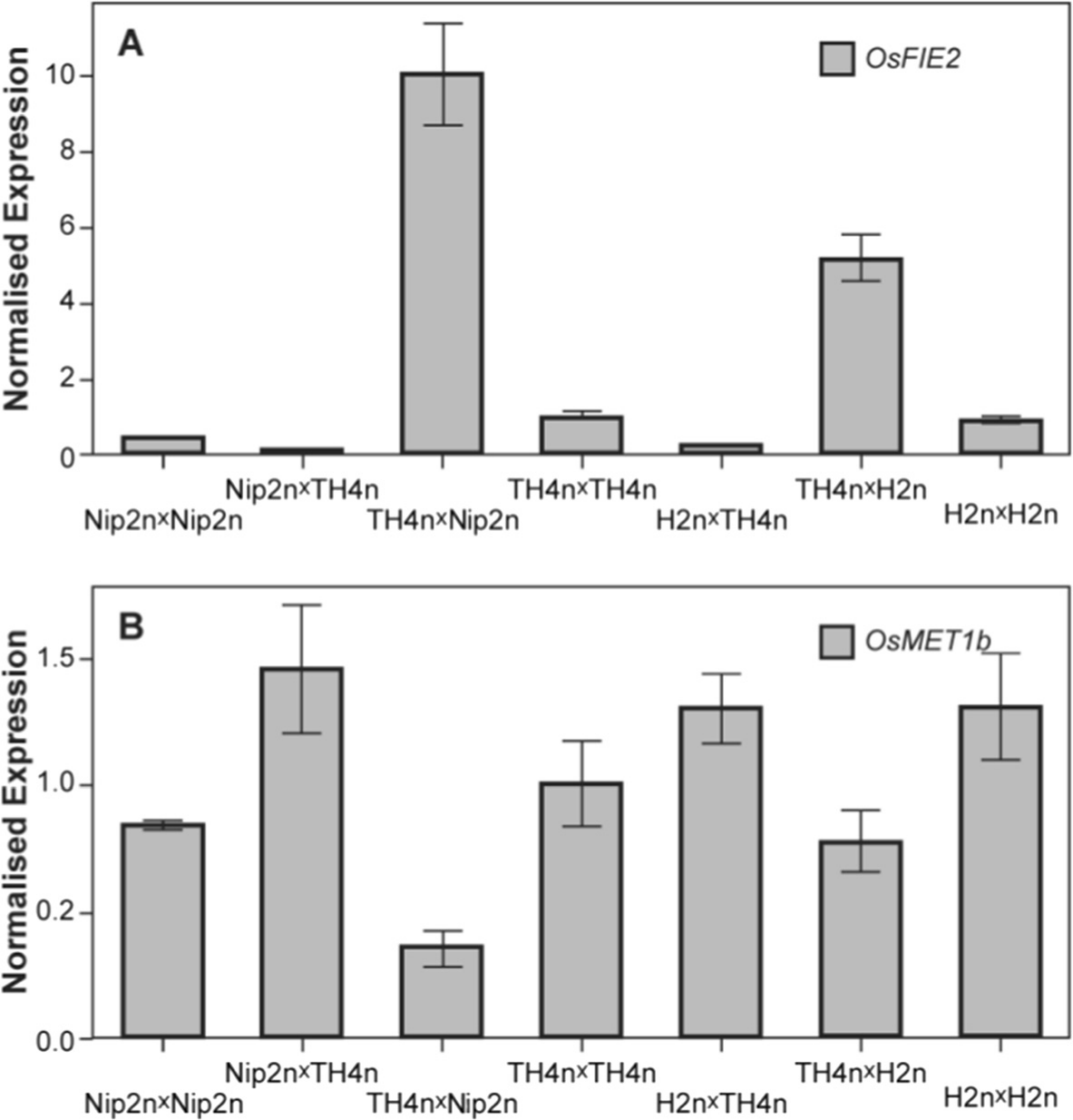
Quantitative RT-PCR of *OsFIE2* and *OsMET1b*. **a** Relative expression levels of *OsFIE2* in 4 DAP endosperm from different crosses. **b** Relative expression level of *OsMET1b* in 4 DAP endosperm from different crosses

Arabidopsis *PRC2* genes and *MET1* play important roles in modulating endosperm cellularization and postzygotic hybridization barriers (Erilova et al. 2009; Kradolfer et al. 2013a; Schatlowski et al. 2014). We investigated the expression levels of *OsFIE2* and *OsMET1b*, two major epigenetic modification genes showing function seed development (Luo et al. 2009; Yamauchi et al. 2009; Nallamilli et al. 2013; Hu et al. 2014; Li et al. 2014) in the endosperm derived from the interploidy crosses and the parents Nip2n, TH4n and H2n (Fig. 3). We used 4 DAP endosperm as the 5 DAP endosperm in unbalanced endosperm started to show degradation and extract 3 DAP endosperm may result in maternal tissue comtamination. In the balanced crosses, *OsFIE2* was expressed similarly between the three parents in the 4 DAP endosperm (Fig. 3). However, *OsFIE2* expression was decreased in the paternal excess cross and elevated in the maternal excess cross, compared to the parents. For *OsMET1b*, expression levels were similar in the parents and the paternal excess crosses, but was slightly decreased in the maternal excess crosses, indicating that the parental genome imbalance influences the expression levels of these two epigenetic regulators.

### Imprinted Genes Were Deregulated in the Interploidy Crosses

The imprinted Arabidopsis *PRC2* genes are deregulated in interploidy crosses (Jullien and Berger 2010). In rice, the only imprinted *PRC2* gene is *OsFIE1* (Luo et al. 2009; 2011). We examined the imprinting status of *OsFIE1* and other imprinted genes in 4 DAP endosperm derived from unbalanced crosses between TH4n and Nip2n. We also used the reciprocal crosses between Nip2n and H2n as controls. The imprinted genes were identified previously using genome-wide transcriptome analysis of crosses between Nip (Japonica) and 93-11, an Indica rice line (Luo et al. 2011). Due to potential maternal contamination when isolating the poorly developed endosperm in the interploidy crosses, we used three endosperm-specific PEGs for this analysis, in addition to 13 MEGs (Luo et al. 2011). Among the 13 MEGs, two (09 g03500 and 11 g27470) became biallelic in the maternal excess cross, two (01 g10080 and 05 g26040) became biallelic in the paternal excess cross, four showing imprinting in the maternal excess cross lost expression in the paternal excess cross, and the remaining five including *OsFIE1* maintained the imprinted expression in both interploidy crosses (Table 2; Additional file 2: Figure S2). For the PEGs, one was imprinted in both interploidy crosses, one became biallelic, and one lost expression in the paternal excess cross (Table 2; Additional file 2: Figure S2). The imprinted genes showing loss of imprinting or expression encode proteins of diverse functions (Table 2; Luo et al. 2011).

**Table 2:** Deregulation of imprinting in interploidy crosses

Gene ID	Bias^a^	Exp^a^	Nip2n × TH4n	TH4n × Nip2n	Function
01 g10080	mat	g	bi	mat	Expressed protein
02 g55560	mat	g	no-exp	mat	Protein phosphatase 2C
05 g26040	mat	en	bi	mat	Pumilio RNA binding protein
05 g34310	mat	en	no-exp	mat	No apical meristem protein
05 g40790	mat	en	mat	mat	CCR4-NOT transcription factor
06 g33640	mat	en	no-exp	mat	CAPIP1
07 g27359	mat	en	mat	mat	Expressed protein
07 g34620	mat	g	no-exp	mat	Expressed protein
07 g42390	mat	en	mat	mat	Intron derived transcripts
08 g04290 (*OsFIE1*)	mat	en	mat	mat	PRC2 member
09 g03500	mat	en	mat	bi	ZOS9-01-C2H2 zinc finger
09 g34880	mat	en	mat	mat	bZIP domain protein
11 g27470	mat	en	mat	bi	Hypothetical protein
07 g17460	pat	en	pat	pat	OsFBL36
10 g04890	pat	en	bi	pat	Expressed protein
12 g08780	pat	en	no-exp	pat	YUCCA like protein

^a^Imprinting and expression status in Luo et al’s (2011) data set (bias > 0.9), and imprinting is confirmed using Nip2n and H2n reciprocal crosses (Additional file 2: Figure S2); exp, expression; mat, maternally expressed; pat, paternally expressed; bi, biallelic; en, endosperm-specific; g, non-tissue specific; H2n, the 2n progenitor of TH4n; See Figure S2 for Sanger sequencing results

## Discussion

We have investigated the effects of parental genome imbalance on seed development in reciprocal interploidy crosses between a 2n Japonica and a 4n Indica rice. The developmental patterns of embryos and endosperm in this study are similar to those observed in a previous study using rice lines of different ploidy levels in the same genetic background (Sekine et al. 2013), suggesting genetic backgrounds in our study did not affect the outcomes of rice interploidy crosses. The increased ploidy levels per se in the triploid seeds are unlikely the direct cause for the disruptive development of embryo and endosperm development in interploidy cross, as polyploid embryo and endosperm with balanced parental genomes are usually viable. Although we cannot rule out the possibility that the relative genome dosages of the two fertilization products to the maternal tissues or even to the female gametophyte (Birchler 2014) cause the seed defects of interploidy cross, it has been speculated that the unbalanced parental genomes cause arrested embryo and disruptive seed development primarily via the unbalanced contributions of parentally expression-biased genes (imprinted genes) and altered endosperm development, as indicated by a detailed genetic analysis in maize and Arabidopsis (Lin 1984; Scott et al. 1998). This notion is also supported by the studies in Arabidopsis where knocking-out endosperm specific imprinted genes or regulators of imprinting can alleviate endosperm abortion and promote seed viability in interploidy crosses (Erilova et al. 2009; Kradolfer et al. 2013a; 2013b; Schatlowski et al. 2014). Our observations support the differential roles of the maternal and paternal genomes in endosperm development (Haig and Westoby 1989; 1991; Dilkes and Comai 2004): excess maternal genome drives the precocious endosperm cellularization and excess paternal genome delays or disrupts cellularization, implying the possible roles of imprinting involved in mediating parent-of-origin effects on seed development in different species.

Our results also suggest that parental genomes have different roles in influencing the translocation of pericarp starch, which is classified as “transitory starch” and plays an important role in grain development (Duffus and Rosie 1973; Sato 1984; Caley et al. 1990). The lack of translocation of pericarp starch coincided with lack of cellularization and starch deposition in the endosperm of the 2n × 4n cross. Interestingly, compared with the 4n × 4n cross, pericarp starch deposition and translocation appears to be the same in the 4n × 2n cross, in which the precocious cellularization and starch deposition occur in endosperm. It is likely that parental genome balance influences pericarp starch relocation via cellularization. The decline of pericarp starch coincides with the rapid expansion of the developing seeds and fast accumulation of starch in the developing endosperm, suggesting the vital role of this transitory starch in grain filling. Degradation of nucellus is required to facilitate the nutrient supply for young embryo and endosperm growth (Yin and Xue 2012). We observed a delayed degradation of the nucellus in seed of the 2n × 4n cross in which precocious cellularization and poor growth of endosperm occur, supporting that degradation of nucellus and endosperm development are linked processes.

Our results also support the idea that endosperm cellularization is a critical developmental transition for embryo differentiation and patterning (Hehenberger et al. 2012). Rice embryo development follows a series of stages identified as zygotic, globular, coleoptiles, juvenile vegetative and maturation (Itoh et al. 2005). The basic plant body plan is established when the embryo reaches the juvenile stage. Our results suggest that the development of endosperm plays a critical role in the differentiation and development of the embryo. Because disruption of endosperm development is the primary cause of seed abortion in parental genome unbalanced crosses (Watkins 1932; Howard 1939; Brink and Cooper 1947; Hakansson 1956; Nishiyama and Inomata 1966; Lin 1984; Ramsey and Schemske 1998; Scott et al. 1998; Gutierrez-Marcos et al. 2003; Stoute et al. 2012; Kradolfer et al. 2013a; Sekine et al. 2013), in the rice paternal excess cross, lack of endosperm cellularization appears hampering further differentiation and development of the embryo, and leads to the embryo arresting at globular stage. In contrast, in the rice maternal excess cross, precocious cellularization leads to the formation of an enlarged embryo showing overgrowth of the scutellum, which may allow the embryo store more nutrients, compensating for the poor reserve of nutrients of the premature endosperm. This suggests that endosperm not only serves as a supplier of nutrients but may also provide signaling molecules at various developmental stages for the differentiation and growth of the embryo. Consistent with this, we have seen better embryo rescue in the maternal excess crosses than in the paternal excess crosses in which embryo arrested early due to lack of endosperm cellularization. Compared with rice embryo, Arabidopsis embryo appears to be more tolerant to interploidy crosses (Scott et al. 1998). The interspecies differences in outcomes of interploidy crosses between rice and Arabidopsis may be related to the intrinsic differences in endosperm development. Rice and Arabidopsis are similar during early endosperm development. However, after cellularization, the endosperm in Arabidopsis disappears and the cotyledons of the embryo replace the nutrient storage function of endosperm. Hence, the endosperm only plays a transient role in supporting embryo development in Arabidopsis. In contrast, rice endosperm continues to proliferate and exists as a major component of the seed. The persistent endosperm is an ideal tissue that provides nutrients and, likely, other signaling molecules for embryo development and germination.

Our results showed that expression of the PRC2 gene *OsFIE2*, a potential imprinting regulator was suppressed in the paternal excess cross, in which endosperm failed to cellularize. This is consistent with the role of H3K27me in rice endosperm development as knocking-down of *OsFIE2* by RNAi results in abnormal endosperm development lacking of cellularization (Nallamilli et al. 2013; Li et al. 2014. Conversely, the elevated expression of *OsFIE2* and slight down-regulation of *OsMET1b* were observed in the maternal excess cross, in which endosperm precociously cellularized. Whether these results imply that the rice *MET1* and PRC2 genes function as the Arabidopsis counterparts to be involved in regulating postzygotic hybridization barriers needs further investigation. Our results also showed loss of imprinting or expression of imprinted genes in the parental genome unbalanced crosses in rice as in Arabidopsis (Erilova et al. 2009; Jullien and Berger 2010), suggesting that deregulation of imprinting is a common phenomenon across species. It appears that more imprinted genes are affected in the paternal excess cross than in the maternal cross. Whether deregulation of imprinted genes causes endosperm abortion, or vice versa in the interploidy crosses remains to be elucidated. Together, our results provide useful clues for further analysis of the roles of imprinted genes and epigenetic factors in endosperm development.

It has been shown that the seed defects in interploidy crosses (Scott et al. 1998; Sekine et al. 2013) are similar to the seed defects in the interspecific crosses (Josefsson et al. 2006; Ishikawa et al. 2011). Increasing the ploidy level of one parent in an interspecific cross was able to improve the viability of hybrid seeds (Johnston et al. 1980; Josefsson et al. 2006), suggesting that there are common mechanisms underlying endosperm abortion in response to both interploidy and interspecific crosses (Haig and Westoby 1991; Schatlowski and Köhler 2012). Imprinting and epigenetic mechanisms regulating imprinting are the promising mechanisms controlling both interspecific and interploidy postzygotic hybridization barrier (Haig and Westoby 1991; Erilova et al. 2009; Kradolfer et al. 2013a; Schatlowski et al. 2014). We reason that low conservation of imprinted genes across species (Luo et al. 2011; Waters et al. 2011; Wolff et al. 2011) contributes to reproductive isolation between related species.

## Conclusion

Our analysis of the differential parental genome dosage effects on the endosperm development in the reciprocal interploidy crosses suggests distinct roles of parental genomes in seed development, with maternal genome functions to promote the endosperm cellularization and paternal genome functions to delay or inhibit cellularization potentially. Starch deposition in endosperm and cellularization regulated by the balance of parental genomes serves as a feedback signal for pericarp starch relocation. Our results support the idea that endosperm cellularization is a critical developmental transition for embryo differentiation and patterning. It is likely that endosperm acts as a sensor in response to post-zygotic incompatibility in interploidy cross via a common mechanism in different species.

## Methods

### Rice Materials and Growth Conditions

Diploid Indica rice Haitan (H2n), a local cultivar was treated with Colchicine following Beachell and Jones (1945) to generate a tetraploid line designated as Tetra-Haitan (TH4n). Japonica rice Nipponbare (Nip2n) was used as a crossing parent. Diploid and tetraploid lines were reciprocally crossed with each other to obtain seeds with different balances of parental genomes.

### Chromosome Counting

Tillers were separated from rice plants. After removing the old roots, the tillers were washed and immersed in clean water to allow the generation of new roots. Roots of 2-3 cm were used for chromosome counting following the methods of Waninge (1965) and Martínez-Gómez et al. (2005).

### Embryo Rescue

Embryos were rescued following the procedure described by Jena and Khush (1984a, b). Embryos of different stages in interploidy or intraploidy crosses were placed on 1/4 MS medium in the dark until root and shoot growth were initiated, and then incubated under light for ~12 days until leaves formed. Rescued seedlings were transferred to full MS liquid medium to promote root growth and then transplanted to soil.

### Microscopy

Pollinated ovaries for serial sectioning were fixed in glutaraldehyde and embedded in Spurr’s resin as described previously (Koltunow et al. 1998). Ovaries were also fixed in FAA and cleared using methyl salicylate in order to examine a greater number of samples, as per Koltunow et al. (2011). All plant material was harvested around 4 pm to avoid the potential fluctuation of starch accumulation during the day. Sections and whole mount ovules were examined using a Zeiss Axioplan microscope and digital images were collected using a Spot II camera. For each stage, at least 8 seeds for each of the four crosses were examined either by clearing and observation under DIC Microscopy, or by sectioning.

### RT-PCR and qRT-PCR and Sequencing

Total RNA was extracted using an RNeasy Mini kit (Qiagen,http://www.qiagen.com/) and treated with RNase-free DNase (Promega,http://www.promega.com/). cDNA was synthesized from 100 ng total mRNA using PRIME Script reverse transcriptase (Takara, http://www.takara-bio.com/) according to the manufacturer’s instructions. Primers used in this study are shown in Additional file 3: Table S1. The TaKaRa SYBR Prenmix Ex Taq™II was used for real time PCR, and data were collected using the BIO-RAD CFX96™ Real-Time System. Relative expression levels from the real-time PCR analysis were normalized against UBIQUITIN and EEF-1A. Bio-Rad CFX Manager V1.6.541.1028 was used to analyse gene expression level.

## Additional files

Additional file 1: Chromosome number counting and seed phenotypes of interploidy crosses. (A-D) Chromosome numbers of root tip cells of Nip2n (A), TH4n (B), 3n plant of Nip2n × TH4n (C), and 3n plant of TH4n × Nip2n (D). (E) Seed phenotypes of balanced and unbalanced crosses. (DOCX 2648 kb) Additional file 2: Sanger sequencing results of selected imprinted genes. (PDF 712 kb) Additional file 3: List of RT-PCR primers. (DOCX 18 kb)
