# Supplementary material for: Parental Genome Imbalance Causes Post-Zygotic Seed Lethality and Deregulates Imprinting in Rice
Source: Rice (N Y). 2016 Aug 27;9(1):43. doi: 10.1186/s12284-016-0115-4 (PMC5002275; doi:10.1186/s12284-016-0115-4)
Supplement: Additional file 1: — Chromosome number counting and seed phenotypes of interploidy crosses. (A-D) Chromosome numbers of root tip cells of Nip2n (A), TH4n (B), 3n plant of Nip2n × TH4n (C), and 3n plant of TH4n × Nip2n (D). (E) Seed phenotypes of balanced and unbalanced crosses. (DOCX 2648 kb) [file 12284_2016_115_MOESM1_ESM.docx]

**Supporting Information**

**
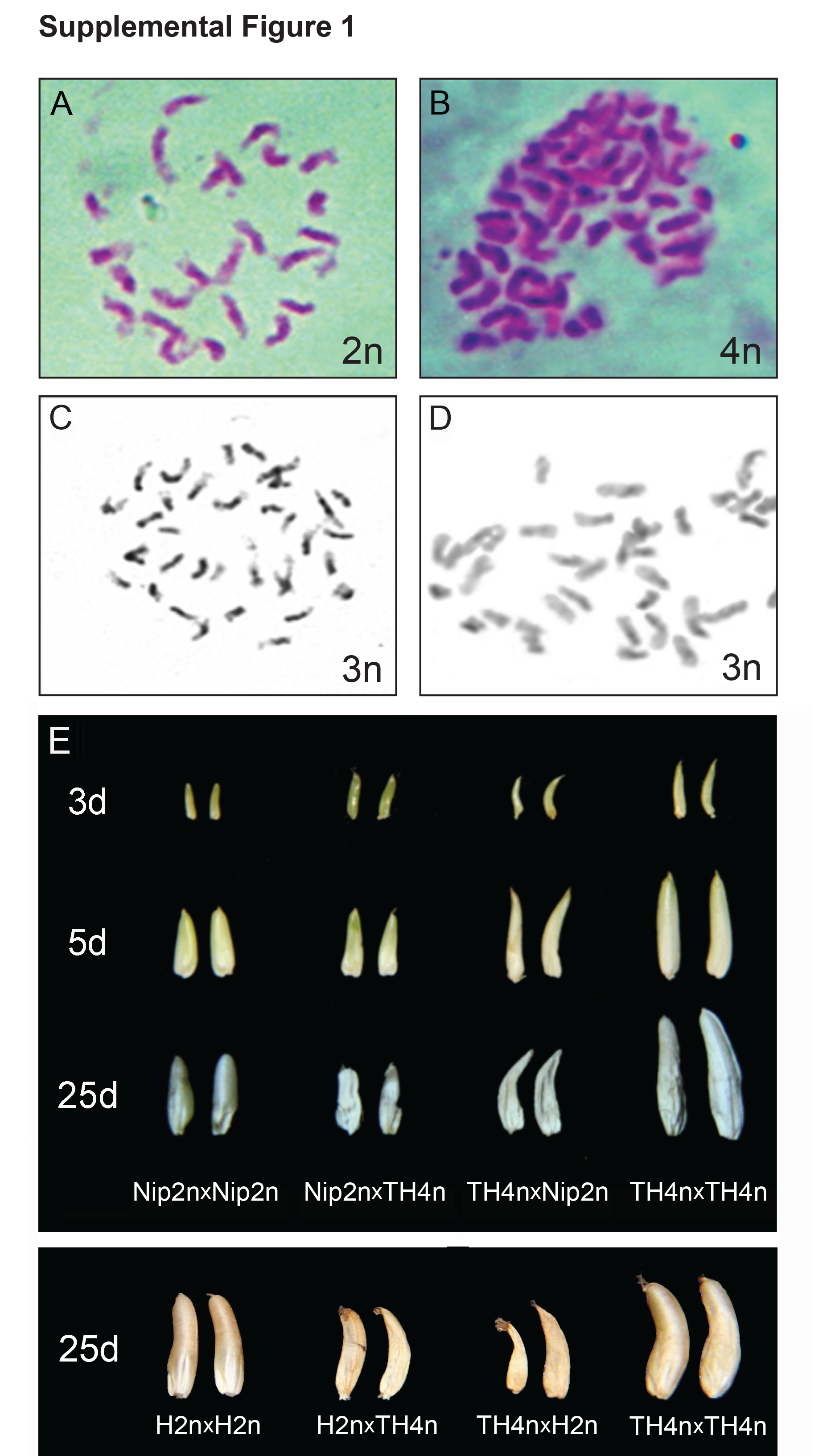
**

**Figure S1. Chromosome number counting and seed phenotypes of interploidy crosses**

(A-D) Chromosome numbers of root tip cells of Nip2n (A), TH4n (B), 3n plant of Nip2n × TH4n (C), and 3n plant of TH4n × Nip2n (D). (E) Seed phenotypes of balanced and unbalanced crosses
