# Supplementary material for: Parental Genome Imbalance Causes Post-Zygotic Seed Lethality and Deregulates Imprinting in Rice
Source: Rice (N Y). 2016 Aug 27;9(1):43. doi: 10.1186/s12284-016-0115-4 (PMC5002275; doi:10.1186/s12284-016-0115-4)
Supplement: Additional file 2: — Sanger sequencing results of selected imprinted genes. (PDF 712 kb) [file 12284_2016_115_MOESM2_ESM.pdf]

Supplemental Figure 2

Gene ID	Bias*	Exp *	Nip2n X TH4n	TH4n X Nip2n	Nip2n X H2n	H2n X Nip2n
01g10080	mat	g	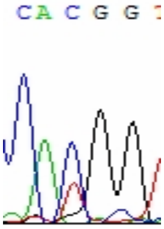	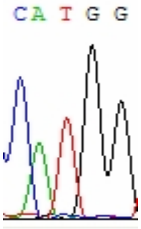	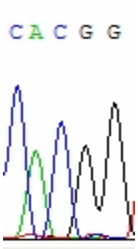	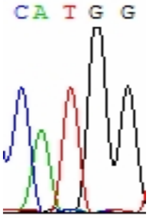
02g55560	mat	g	no-exp	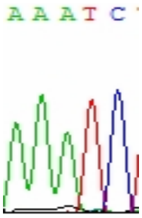	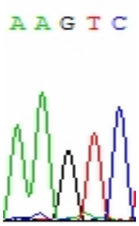	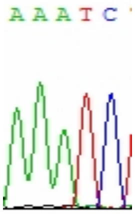
05g26040	mat	en	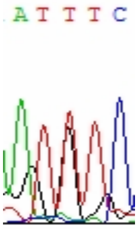	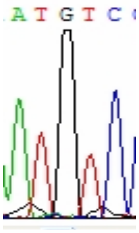	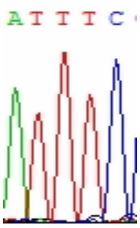	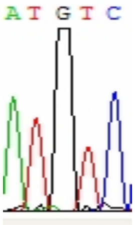
05g34310	mat	en	no-exp	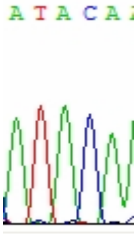	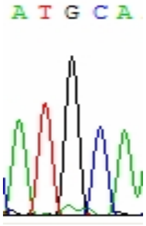	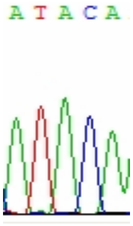
05g40790	mat	en	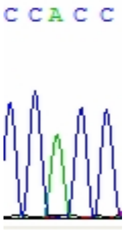	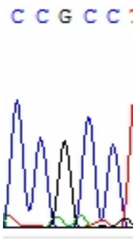	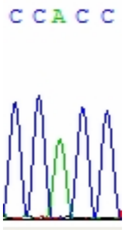	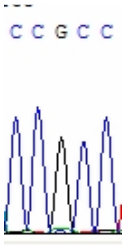

Gene ID	Bias*	Exp *	Nip2n X TH4n	TH4n X Nip2n	Nip2n X H2n	H2n X Nip2n
06g33640	mat	en	no-exp	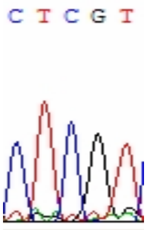	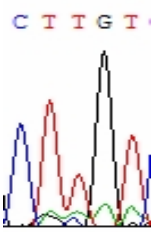	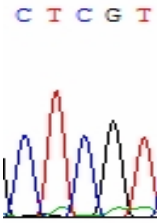
07g27359	mat	en	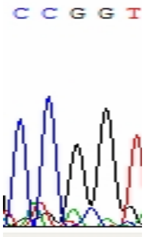	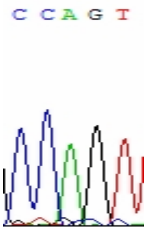	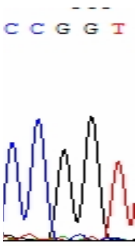	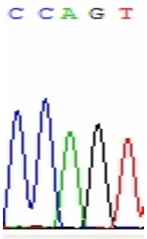
07g34620	mat	g	no-exp	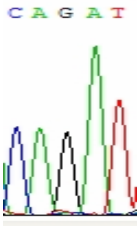	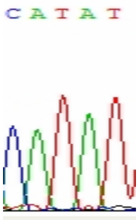	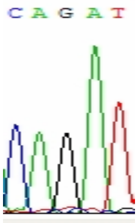
07g42390	mat	en	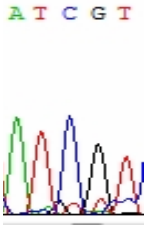	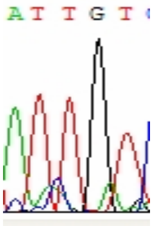	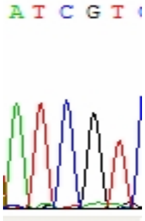	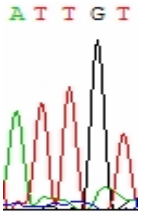
08g04290	mat	en	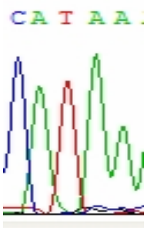	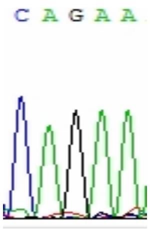	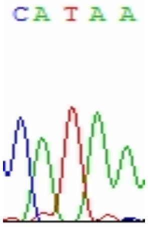	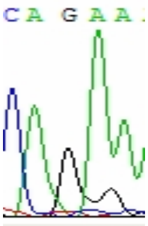

Gene ID	Bias*	Exp *	Nip2n X TH4n	TH4n X Nip2n	Nip2n X H2n	H2n X Nip2n
09g03500	mat	en	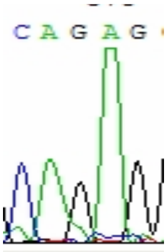	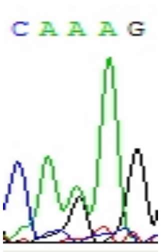	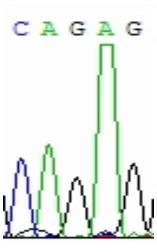	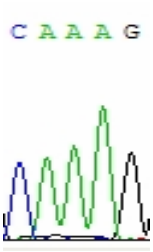
09g34880	mat	en	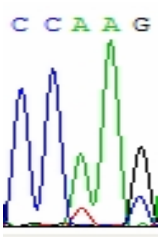	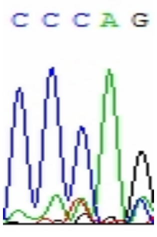	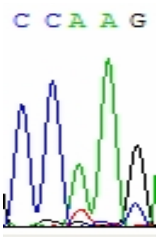	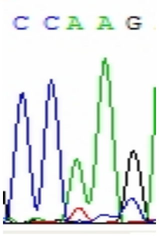
11g27470	mat	en	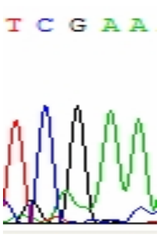	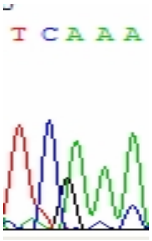	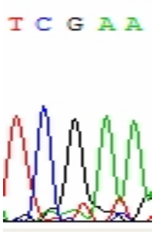	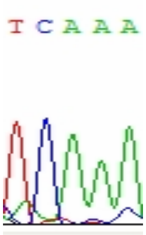
07g17460	pat	en	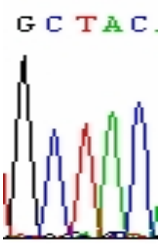	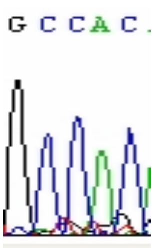	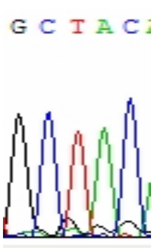	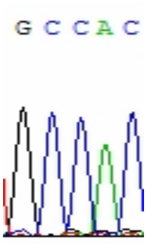
10g04890	pat	en	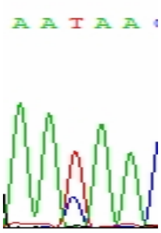	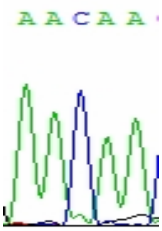	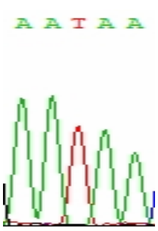	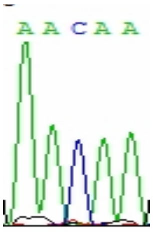

<table>
<tr><th>Gene ID</th><th>Bias*</th><th>Exp *</th><th>Nip2n X TH4n</th><th>TH4n X Nip2n</th><th>Nip2n X H2n</th><th>H2n X Nip2n</th></tr>
<tr><td>12g08780</td><td>pat</td><td>en</td><td>no-ex</td><td>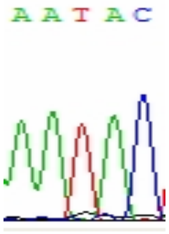
</td><td>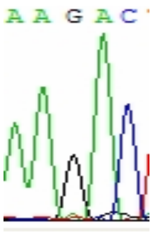
</td><td>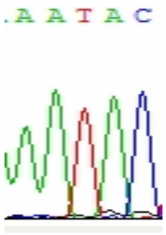
</td></tr>
</table>

*Imprinting status in Luo et al’s (2011) data set; exp expression; Mat maternally expressed; pat paternally expressed; Bi biallelic; en endosperm-specific; g non-tissue specific.
