# Supplementary material for: Parental Genome Imbalance Causes Post-Zygotic Seed Lethality and Deregulates Imprinting in Rice
Source: Rice (N Y). 2016 Aug 27;9(1):43. doi: 10.1186/s12284-016-0115-4 (PMC5002275; doi:10.1186/s12284-016-0115-4)
Supplement: Additional file 3: — List of RT-PCR primers. (DOCX 18 kb) [file 12284_2016_115_MOESM3_ESM.docx]

**Table S1. List of RT-PCR primers**

| Locus ID | Primer Sequence (5' → 3') |
| --- | --- |
| *Ubi* | F：CCAAGAAGAAGATCAAGAAC |
|  | R：GATAACAACGGAAGCATAAAAGTC |
| *EEF-1* | F：TTTCACTCTTGGTGTGAAGCAGAT |
|  | R：GACTTCCTTCACGATTTCATCGTAA |
| LOC_Os01g10080 | F：GGCTAAAGAAGGATGCGGATA |
|  | R：CACCAACGCACTAAGCTCAA |
| LOC_Os04g39560 | F：CTCTCACCGCGTGGATCT |
|  | R：CCTAGCTCAGCAGCTCATCC |
| LOC_Os05g40790 | F：GTGGTGCCAGATCAAACCTT |
|  | R：AGGAACCCCTTGGTGCTAGT |
| LOC_Os02g55560 | F：AGAGAGGCTCGATTTTGGTG |
|  | R：CGACCTAGCCTGCTTGAAAT |
| LOC_Os03g27450 | F：AGGCCGTACAAGTTTCAAGC |
|  | R：ACCACCCAATCTATGCCAGA |
| LOC_Os06g40490 | F：CTGCTCTTCTGCCTCCTCCT |
|  | R：GGAGAAGGGATGTTGTTTGC |
| LOC_Os07g17460 | F：CGACCTTGATGACGGCTATT |
|  | R：TCTTGATGCTCGACACTGGT |
| LOC_Os09g03090 | F：CCATCAGGAATGTCTACTGAGC |
|  | R：GGTGCTGCTTGATGGAAAGT |
| LOC_Os07g34620 | F：CGCGAAGATCCTCTTGAAAC |
|  | R：TGTAGTCCGTGACGTCGTTG |
| LOC_Os07g27359 | F：TGCAAGACACTGATCATCCAA |
|  | R：TTTGGATGCGTGTTAATCCA |
| LOC_Os04g08570 | F：CTTGGGAGGGGTAGATCCAT |
|  | R：ATGCATGCACCTAGACGTTG |
| LOC_Os04g42250 | F：GCCTCGCTTTGGTCTTCAT |
|  | R：ACCGTGATCGCCATCTTC |
| LOC_Os12g08780 | F：CAGGCATGGAGATTGCCTAT |
|  | R：ATCCCCATGTGAATCAGCTC |
| LOC_Os01g42270 | F：GACAACAGCAAAAGCCTTCC |
|  | R：AAAAAGAAAGCCACCAGGA |
| LOC_Os05g34310 | F：CGCGGATGTCGAAATATACA |
|  | R：GGTACCTGTGGTCGCGTAGT |
| LOC_Os07g12490 | F：GTACGCATGGAGGTTCCAGT |
|  | R：ATGCTTCAACCCGCTTTAGA |
| LOC_Os11g07910 | F：CCATTGAAAGGTCATATTGG |
|  | R：GTTGTTTTTGTCAGTCTCACC |
| LOC_Os07g08500 | F：GCGGTGTTTGTGGATAACTG |
| （*OsMET1b*） | R：TCAGCAGCCTCAGAAGTTGA |
| LOC_Os08g04290 | F：TACAGACCAGCCCTCCTGTT |
| (*OsFIE2*) | R：GCCATATGTTGCCATCCTCT |
